# Grand Challenge in Adrenal Endocrinology: Is the Legacy of the Past a Challenge for the Future of Precision Medicine?

**DOI:** 10.3389/fendo.2021.747006

**Published:** 2021-09-03

**Authors:** Iacopo Chiodini, Luigi Gennari

**Affiliations:** ^1^Department of Endocrine and Metabolic Diseases, IRCCS, Istituto Auxologico Italiano, Milan, Italy; ^2^Department of Medical Biotechnology and Translational Medicine, University of Milan, Milan, Italy; ^3^Department of Medicine, Surgery and Neurosciences, University of Siena, Siena, Italy

**Keywords:** glucocorticoid, aldosterone, glucocorticoid receptors, cortisol, 11β-hydroxysteroid dehydrogenase, paraganglioma-pheochromocytoma

## Introduction

In recent years, more and more evidence has emerged on the deleterious systemic effects arising from adrenal hormone alterations, even if mild and/or asymptomatic. On the other hand, an increased prevalence of adrenal diseases has been reported in patients without signs and symptoms specific of adrenal disorders but affected by chronic diseases or conditions (ie diabetes, hypertension, osteoporosis, depression, fibromyalgia, hypotension) that are often misdiagnosed as ‘idiopathic’ but which should instead be attributed to observed alterations in adrenal function (1–7). Such an expansion in knowledge of the role of adrenal hormones in the pathophysiology of common human diseases is in accordance with well-known evidence arising from studies in mammals that adrenal hormones influence several functions, including different metabolic pathways, immune responses, cognition, circadian rhythms, saline homeostasis, blood pressure control and stress response (8). However, more recently, not only has the degree of secretion of adrenal hormones been suggested, but also their metabolism and sensitivity within peripheral target tissues have been suggested to play a role in the development of chronic disorders such as hypertension, diabetes, osteoporosis, and mood disorders, even in subjects with biochemically normal adrenal function (9–18).

Therefore, since occult adrenal hormone dysfunction can occur in some categories of patients, recent research has focused on the screening of adrenal disorders among subjects affected by bone fragility, hypertension, and type 2 diabetes (2, 4). However, the aftermath of a wide screening is the finding of false positive results, with potential economic and psychological consequences for health care systems and the patients, respectively (19). Thus, optimizing the accuracy of screening tests has been advocated in order to avoid overdiagnosing and to individuate true positive cases. In this sense, recent findings that various adrenal disorders have a genetic basis and that genetic variants may predispose to the development of these diseases could lead to a better focus on providing screening tests to the individuals at risk (20–23).

The path toward precision medicine in adrenal endocrinology has just begun (24) and the future challenges for its further development should be addressed to the following questions: i) when to suspect and who to screen for adrenal dysfunctions; ii) what and how to measure to reliably diagnose adrenal diseases; iii) how to search for adrenal hormone secreting lesions; iv) how and what can cure patients with adrenal disorders.

## When to Suspect and Who to Screen for Adrenal Diseases

**Figure 1:**
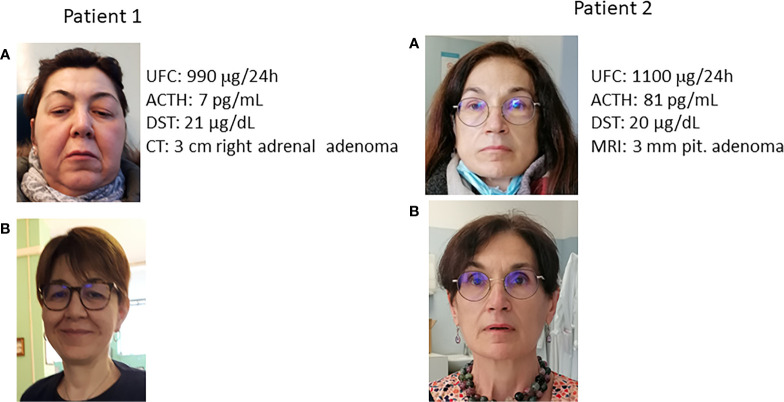
Clinically overt Cushing syndrome and occult hypercortisolism. **(A)** at diagnosis; **(B)** after cure. UFC: urinary free cortisol (normal values 20-137 μg/24h); ACTH: adrenocorticotroph hormone (normal values 5-55 pg/mL); DST: cortisol after 1 mg overnight dexamethasone suppression test (normal values <1.8 μg/dL); MRI: Magnetic Resonance Imaging; CT computed tomography. Patient 1: Clinically overt Cushing syndrome. The patient was referred for the clinical picture and the presence of spontaneous vertebral fracture. Note the typical facies plethorica. Patient 2: Occult hypercortisolism. The patient was referred for resistant hypertension. It is worth noting the absence of facies plethorica. In the photo before cure the possible presence of a slight swelling in the jaws is evident only after comparison with the photo after cure. After normalization of cortisol secretion, blood pressure levels normalized and no antihypertensive treatment was needed anymore. It is of note that the degree of cortisol hypersecretion was similar between the two patients (reflected by UFC and DST levels), despite the completely different clinical picture.

In the past, screening for disorders due to altered adrenal hormone secretion was reserved for patients with specific signs and symptoms, such as plethora, striae rubrae, buffalo hump, and hypertricosis (patient 1, **Figure 1**). This traditional approach prevails in the practices of many physicians since the idea that individuals without overt signs and/or symptoms of adrenal dysfunction could be affected by adrenal disorders is scarcely accepted by the vast majority of experts in the field. However, in recent years several reports have described the presence of occult alterations in adrenal hormone secretion in patients with nonspecific conditions (such as hypertension, osteoporosis, diabetes, mood disorders) or nonspecific symptoms (fatigue, anorexia, weight loss, hypotension, hyponatremia, and hyperkalemia) (4, 25) (patients 2, **Figure 1**). This difficulty in turning the observation point upside down has caused that, to date, no widely accepted guidelines are available in order to improve the diagnosis of occult adrenal dysfunction. As a consequence, scarce data are available on the true prevalence of adrenal causes of some chronic diseases and conditions, such as the above-mentioned ones.

For example, the available guidelines consider it mandatory to screen for occult adrenal hormone hypersecretion in hypertensive patients in the presence of specific characteristics of hypertension even in the absence of typical symptoms of adrenal disorders (5, 26, 27). Notwithstanding these indications, few studies have been conducted to date to assess the prevalence of hypercortisolism in hypertensive patients. Importantly, in patients with hypercortisolism, normalizing cortisol secretion generally improves or even normalizes blood pressure (28). In contrast, no guidelines have been provided for the detection of hypercortisolism in patients with diabetes or osteoporosis, despite several studies reporting an unexpected increased prevalence of subtle excess cortisol excess in these patients (2, 29). Currently, we only have some expert opinions suggesting that patients with poorly controlled diabetes or complicated controlled diabetes and hypertension, as well as patients with unexplainable bone fragility or severe osteoporosis, should be screened for hypercortisolism (2, 30, 31). Consistent with the hypothesis of a current underestimation of the number of patients with adrenal diseases, the incidence of paraganglioma (PGL) has increased 4.8 times from 1977 to 2015, which is likely due to its recognition in patients with PGL found in small incidentally discovered adrenal masses and few or no paroxysmal symptoms (22). This finding suggests that current guidelines, based on old data, may not be adequate to detect patients with PGL seen in daily clinical care today. Likewise, despite some expert opinions, no guidelines suggest screening for primary adrenal insufficiency in cases of asthenia, weight loss, dehydration, hypotension, fever, abdominal pains, nausea, and hyperpigmentation or patients with type 1 diabetes, autoimmune thyroid disease, unexplained collapse, hypotension, vomiting or diarrhoea (4).

More recently, adrenals, and in particular the hypothalamic-pituitary-adrenal axis (HPA) have been identified as a likely target of immune therapies and checkpoint inhibitor therapies in patients with cancers (32). Most patients present with nonspecific symptoms, such as fatigue, which can be erroneously attributed to the malignant disease itself. However, up to 6% of patients treated with these drugs may experience hypophysitis and possible disruption of the HPA axis or primary adrenal insufficiency (32), which are two well-known life-threatening conditions. Therefore, in the future, screening for adrenal or HPA activity disorders should become a routine practice for oncologists using these therapies, even in the absence of specific signs and symptoms for adrenal insufficiency.

## What and How to Measure

Nowadays, the diagnosis of adrenal diseases is somehow hindered by the cornerstones achieved in the past (e.g. concerning the metabolites to be measured, the methods to use, and the cut-offs to apply). Indeed, what we have inherited from old literature, seems to quickly become a legacy to overcome rather than supporting pillars to rely on. For example, several studies suggest that the cutoff of cortisol after 1 mg overnight dexamethasone suppression test to diagnose hypercortisolism (i.e. 1.8 μg/dL) might not be sufficiently sensitive and that lower cut-offs might be adopted (33–36). Paradoxically, some guidelines still advocate even higher cut-off points for diagnosing autonomous cortisol excess (37). Furthermore, nowadays, several pieces of evidence point to the limited reliability of some methods for measuring cortisol secretion (i.e. salivary cortisol and urinary free cortisol) in patients with possible occult hypercortisolism (38), but these tests are still used due to the low sensitivity of endocrinologists for diagnosing subtle excess cortisol. As a consequence, many patients with hypercortisolism have been left undiagnosed until now (2). Similarly, the recent introduction of more specific cortisol assays results in lower cortisol concentrations and this may increase the number of patients diagnosed with adrenal insufficiency. The medical community should quickly adopt the new criteria, as this could lead to misdiagnosis and overtreatment in patients suspected of hypocortisolism (39).

The limited specificity due to cross-reaction with endogenous steroids of the traditionally used cortisol immunoassays results in limited precision and high variability between laboratories. Recently, liquid chromatography tandem mass spectrometry (LC-MS/MS) measurements of cortisol in saliva and urine have been shown to eliminate the influence of the cross-reactivity with cortisone and other steroids, thus providing higher specificity as compared with immunoassays. In patients with suspected hypercortisolism, the use of LC-MS/MS increases specificity, thus reducing the number of false positive results. On the other hand, since in patients with hypercortisolism the clinical picture may also depend on the secretion of steroid precursors, paradoxically, the sensitivity for Cushing syndrome detection may be similar, if not higher, for many immunoassays already in use than for LC-MS/MS. However, this latter method could be useful in the suspicion of factitious hypercortisolism, in establishing whether the achieved level of dexamethasone is appropriate for DST, in the diagnosis of polycystic ovarian syndrome (40) and in medically treated patients with Cushing’s syndrome (41). However, again in these patients, measuring cortisol and its metabolites by immunoassay could be more representative of real glucocorticoid (GC) secretion and, possibly more predictive of clinical outcomes, than measuring only pure cortisol levels by LC-MS/MS. Therefore, the use of LC-MS/MS measurement of a comprehensive steroid profile, rather than cortisol alone, could be proposed for the detection of subtle excess GC in patients with incidentally discovered adrenal masses (42, 43). In addition, 11-deoxycortisol in serum or urine could become a sensitive marker of residual adrenal function in the future. Indeed, thanks to the use of the LC-MS/MS measure of the steroid metabolome, residual adrenal function has been suggested to be present in up to 30% of patients with Addison’s disease (44). Finally, the possibility of measuring cortisol and cortisone with LC-MS/MS can consent to evaluate the degree of peripheral activation of cortisone in cortisol, which could become an important marker to evaluate the cortisol milieu in the individual patient (14, 45). Other methods for measuring cortisol will probably be available, such as surface-enhanced Raman spectroscopy, in addition to immunoassay, which has recently been suggested to be even better than LC-MS/MS in terms of sensitivity for *in situ* monitoring of human stress levels and cortisol-related disorders (46).

Long-standing exposure to an excess or deficiency of glucocorticoids is a likely explanation for the lack of signs and symptoms in more and more patients with hypercortisolism or hypocortisolism, respectively. Along with the advantages of being noninvasive methods that do not require special storage conditions, the use of hair or nail samples to assess cortisol concentration has the important advantage of giving retrospective information about the accumulation of cortisol, allowing for the evaluation of cortisol secretion in the long term. Although to date, this information is available mainly on stress disorders, in the future, the use of hair and nail cortisol determination could become important even for the evaluation of patients with hypercortisolism or hypocortisolism (47–49).

The genetic approach to the diagnosis of adrenal disease will probably be used more and more in the future. For example, evaluating the genetic variants of the GC receptor (18) and the 11β-hydroxysteroid dehydrogenase enzymes (50) could consent to predict the risk of being affected by some cortisol-related disorders (51). Already now, the search for genetic mutations in patients with Addison’s disease or PGL has become extremely important for possible implications and genetic counseling (52–55) and similar data are coming even regarding functioning adrenocortical adenomas (21, 55). In patients with aldosterone-producing adenoma, associations have also been proposed between histology, genotype, race, gender, and clinical characteristics, suggesting the need to consider several aspects together to improve the clinical management of these patients (56, 57).

## How to Search

An everyday challenge for adrenal endocrinologists is to assess: i) when to functionally characterize an adrenal mass (particularly if incidentally found); ii) how to detect very tiny lesions or to distinguish between benign and malignant adrenal lesions; and iii) how to find an extra-adrenal source of an excess adrenal hormone. These difficulties are common challenges in patients with suspected adrenocortical carcinoma (ACC), extra-adrenal or metastatic PGL, or primary aldosteronism (PA). We have become used to characterizing adrenal tumors by measuring Hounsfield units and contrast wash out by computed tomography (CT) and chemical shift with magnetic resonance imaging (MRI), respectively, in selected patients. In case of doubt about possible malignancy, 18F-fluorodeoxyglucose (FDG) positron emission tomography (18FDG-PET) may be helpful, as adrenal carcinomas are generally, but not invariably, 18FDG-PET positive. Therefore, new radiotracers are needed for a more accurate diagnosis, possibly based on the new acquisitions on metabolomics (58).

Recently, the European Association of Nuclear Medicine has provided indications on the use of CT/MRI or [68Ga] Ga-DOTA-SSA PET/CT or [18F] FDOPA to assess an adrenal or extra-adrenal mass suspicious for adrenal PGL. These experts agreed on the need to choose these methods based on genetic characterization (i.e. HIF2A/VHL/MAX, SDHB mutations) (59, 60). Finally, 11C-metomidate, an inhibitor of 11-β-hydroxylase, has been found to be highly specific for adrenocortical masses and a potentially useful PET tracer for the identification of PA and ACC. These recent advances can provide two lessons to be learned. First, in the future, the introduction of new radiopharmaceuticals, especially those with theranostic potential, together with a better genetic characterization of adrenal tumors will change the landscape and will enable individualized treatment schemes to achieve higher responses while reducing toxicity. Second, obtaining consensus and setting up networks among experts will allow patients to discuss their clinical cases in multidisciplinary teams for better clinical management.

## How and What to Cure

Historically, the cure of adrenal disorders includes two equally important issues: the treatment of hormonal alteration and the treatment of adrenal disease-related comorbidities. The new challenge is to be aware that these issues are important in all patients with adrenal disorders, not only in severe cases, and that the coexistence of certain risk factors or complications can influence treatment decisions (61).

For example, surgery remains the definitive cure for patients with unilateral adrenal functioning tumor, but today more and more patients are diagnosed with bilateral adrenal lesions and/or are not feasible for surgical procedures due to advancing age or high risk of surgical complications. These patients need to be treated medically. In patients with adrenal hypercortisolism, as now, medical therapy includes adrenal steroidogenesis inhibiting agents (eg ketoconazole, metyrapone, mitotane, etomidate) and GC receptor blocker (mifepristone). New steroidogenesis inhibitors (i.e., osilodrostat) and other GC receptor antagonists with less adverse effects compared to mifepristone are in clinical trials (relacorilant) and a new drug is being developed, ATR-101, which exerts its action on cholesterol acyltransferase 1, a transmembrane enzyme involved in cholesterol metabolism (62, 63). Since the steroidogenesis inhibitors act on adrenal enzymes on multiple levels with different selectivity, potency, and onset of action, sequential/combination schemes may be considered in the future in some patients. Here, the first challenge is to choose the best drug for individual patients. Second, steroidogenesis inhibitors are titrated to normalize urinary free cortisol, but with GC receptor antagonists, as now, it is not possible to biochemically evaluate the drug effect, which can only be assessed clinically. Third, the goal of controlling hormonal hypersecretion must be achieved by avoiding the risk of overtreatment, which may be as deleterious as the disease itself. This is a common problem even in patients with congenital adrenal hyperplasia, in whom the amount of GC used to inhibit ACTH could lead to overtreatment. In these patients, the combination of GC at lower doses with nonsteroidal and selective corticotroph releasing hormone receptor antagonists could prove to be effective in controlling androgen secretion (64). Finally, increasing the selectivity of steroidogenesis enzymes is another goal in the field of adrenal endocrinology. In PA, new non-steroidal mineral-corticoid antagonists, selectively targeting aldosterone synthesis, which are more specific than spironolactone and more potent than eplerenone, are currently under development. Even for PA patients, future challenges will be to find new targets for medical therapy based on genetic studies. Based on the discovery of recurrent mutations in genes encoding ion channels in PA, new perspectives are opening for targeted therapy for patients carrying specific mutations, and, paradoxically, some old drugs that act on calcium and sodium channels, such as macrolide antibiotics, verapamil, and amiloride, could live a new life (57).

The challenge of preselecting patients to personalize therapy must be played particularly in patients with advanced adrenal cancer (ACC) or with metastatic PGL. For ACC, chemotherapy has limited efficacy and the clinical utility of available targeted therapies alone or in combination with immune checkpoint inhibitors has been shown to be scarce. However, some case reports suggest that certain patients have experienced clinical benefits with these therapies. Furthermore, it is possible that in these patients elevated levels of GC limit the efficacy of these agents. Here, the challenge is to develop biomarkers that predict which patient would have the greatest benefit to immunotherapies (65). For patients with metastatic PGL, therapy with 131I-metaiodobenzylguanidine is already used in selected cases. As discussed above, the genetic background of patients with PLG is complex, but the genetic expression profile could be useful to guide the choice of radiopharmaceutical treatment (53).

Despite the recent development of new modes of GC delivery that could improve quality of life in some patients with adrenal insufficiency, and although further therapy options will probably be available in the future, current treatment is not curative, but simply attempts to replace physiological cortisol concentrations. Therefore, the real challenge would be stopping and possibly reversing autoimmune destruction. Studies using rituximab or using chronic stimulation by adrenocortical releasing hormone stimulation have been shown to reactivate adrenocortical function in some patients (44). The production of autologous stem cells and transplantation of adrenocortical tissue are other promising approaches. Again, in the future, a deeper understanding of the pathophysiology of adrenal insufficiency could contribute to the development of tissue engineering strategies and the delivery of regenerative therapies.

Research should focus on the therapy of adrenal disorders and be directed to consider the possible and previously not considered consequences of adrenal hormone alterations. Recently, the issue of quality of life (QoL) and long-term psychosocial consequences have received increased attention. Patients with both hypercortisolism (whatever the degree) (66, 67) and hypocortisolism (54) may present with reduced quality of life and impaired cognitive function. The idea would be to develop patient-centred studies on QoL and psychological status that could be as informative as disease-centred ones (68). Finally, new insights on the expression of adrenal hormone receptors in various cells may suggest that some new organs or tissues could be possible targets of adrenal hormone-related comorbidities. For example, osteoporosis and hyperparathyroidism have recently been suggested to be a possible complication of aldosterone excess, in keeping with the presence of mineralcorticoid receptors on the surface of parathyroid cell surface (69).

## Conclusions

The pathophysiology of adrenal diseases represents a challenging and still poorly investigated topic.

The field of research and new issues have to be better explored in the future. For example, some recent data point to the possible role of gut microbioma in modulating cortisol metabolism, due to the presence of 11β-hydroxysteroid dehydrogenase activity that plays a pivotal role in glucocorticoid metabolism. As new 11-hydroxysteroid dehydrogenase microbial 11β-hydroxysteroid dehydrogenase enzymes are continually being discovered, a new mechanistic study of their impacts in disease models could be developed (70). Likewise, the role of aberrant G-protein coupled hormone receptors (GPCR) in the pathogenesis of adrenal tumors has been investigated for many years. However, it is still a matter of debate whether aberrant GPCR expression is the initiating event or a consequence of the proliferative cascade underlying adrenal hyperplasia or tumorigenesis. Animal models suggest that a single inappropriate expression of a nonmutated GPCR gene is sufficient to initiate the process that leads to the formation of a benign adrenocortical lesion. On the other hand, other data support the idea that aberrant expression of one or more GPCRs and their ligands in adrenal tissues is a secondary rather than a primary event (71). Interestingly, very recent evidence from animal studies suggests that chronic stress in early life can induce persistent up-regulation of the HPA axis that generates endocrine, metabolic, and somatic alterations similar to those found in ACTH-dependent human ACTH-dependent hypercortisolism (72).

The relationship between the activity of the HPA axis and stress-related diseases and mood disorders and their reciprocal influence has been studied in the past and is likely to continue to be studied in the future (7, 11, 73). This represents an interesting model of how the degree of cortisol secretion (although within the normal range) can contribute to the pathogenesis of chronic diseases. For example, some recent data suggest that, even in eucortisolemic patients, the individual degree of exposure to cortisol (also called cortisol milieu) may influence cardiovascular risk and that in obese eucortisolemic patients, the degree of cortisol secretion is an independent predictor of diabetes and hypertension (16). Furthermore, increased GC sensitivity due to sensitizing polymorphisms of GC receptors was shown to be associated with worse glycometabolic and lipid profiles in subjects without hypercortisolism. Likewise, in eucortisolemic patients with diabetes, modulation of 11bHSD activity was found to improve diabetes control. Finally, a growing body of evidence suggests a positive relationship between physiological, yet higher, urinary cortisol levels and cardiovascular risk (25).

The field of adrenal endocrinology is expected to have a bright future, provided that we can change our approaches, leaving the inherited dogma that adrenal diseases are rare disorders, while increasing awareness that alterations in adrenal hormones could be found in several chronic disorders, also contributing to their pathogenesis.

## Ethics Statement

Informed consent was obtained from patients for the publication of their photos and biochemical and clinical data.

## Author Contributions

LG: conceptualization, writing—original draft, and writing—review and editing. IC: conceptualization, writing—original draft, and writing—review and editing. All authors contributed to the article and approved the submitted version.

## Funding

This study has been partially supported by the Istituto Auxologico Italiano, IRCCS (Grant 05C921 - PRECOR Study).

## Conflict of Interest

LG has no financial relationships with any organization that might have an interest in the submitted work and no other relationships or activities that could appear to have influenced the submitted work.

IC is an investigator in relacorilant studies (Corcept Therapeutics) in patients with hypercortisolism and received consulting fees from Corcept Therapeutics and HRA Pharma.

## Publisher’s Note

All claims expressed in this article are solely those of the authors and do not necessarily represent those of their affiliated organizations, or those of the publisher, the editors and the reviewers. Any product that may be evaluated in this article, or claim that may be made by its manufacturer, is not guaranteed or endorsed by the publisher.
